# Supplementary material for: Sclerochronological characteristics of Orbicella faveolata in Cayo Arenas, a remote coral reef from the Gulf of Mexico
Source: PLoS One. 2023 Nov 14;18(11):e0293802. doi: 10.1371/journal.pone.0293802 (PMC10645327; doi:10.1371/journal.pone.0293802)
Supplement: Attachment — Submitted filename: PONE-D-23-05903_Review.docx [file pone.0293802.s002.docx]

In this manuscript the authors obtain growth skeletal parameters (linear extension, density and calcification ) from five different colonies of Orbicella spp. These cores were all collected from the remote reef of Cayo Arenas. Growth parameters were obtained from 2-dimensional x-rays following previously published methods. These growth data were coupled to annual SST (obtained through remote sensing) to evaluate how SST changes over time might be affecting growth of this massive reef-building coral. The authors found a significant negative linear relationship between maximum annual SST and coral calcification and how negative trends in skeletal growth have been observed from 1998 onwards. Importantly, the periods of time where the growth change rate is larger seem to align with periods with abrupt positive thermal anomalies.

This study constitutes the first reported dataset from the reef-building Orbicella from Cayo Arenas, and its results are of importance to further understand how different corals respond to climate change and its thermal thresholds.

As such, I recommend this manuscript to be accepted after major revisions have been addressed as detailed below. In addition to these changes, I would recommend to thoroughly examine both the grammar and the narrative of the manuscript, as at times it feels hard to follow the authors on their science.

**Major changes:**

Abstract

Line 38: [there] will be [ sic] is a prediction and needs to be supported by evidence/references. This sentence does not make sense. Rewrite.

Introduction

Line 62: Add references for studies that look into stress bands and past climate events.

Line 65: References to this sentence (Sclerochronological records from corals are important to understand the effect of climate change on coral calcification) are not entirely adequate. There are other important papers than can be cited here and that have been omitted from the literature review. For example, Cantin et al. 2010 (DOI: 10.1126/science.1190182) or Cooper et al. 2012 (DOI: 10.1126/science.1214570) among many other that use coral growth records coupled to temperature data to explore past changes in calcification due to thermal stress. A more thorough review would be useful for the reader, not only with data from the Atlantic/Caribbean but also from other massive species from the Pacific. This can help contextualise how ocean warming affecting corals is a global effect, and this study adds evidence to attests this.

In the introduction, the second and third paragraphs need to be re-structured. In Line 59 (Second paragraph) there is a hint at the existence of “stress bands” as a result of coral sensitivity. This seems unconnected to the rest of the paragraph. Furthermore, in the third paragraph, from Line 68 it focuses on thermal stress and how this leads to stress bands. Instead, it might be easier from the reader perspective to follow a structure similar to: i)Coral calcification is important to maintain net reef accretion. Calcification records can aid in reef management efforts (the first paragraph of the introduction as it is); ii) Coral growth is sensitive to several environmental factors, with temperature being the most important variable. Sensitivity of the coral symbiont to this variable produces changes in coral growth. This is the basis for coral sclerochronology; iii) Skeletal formation is characterised by annual couplets of high-density and low-density bands (the base of the second paragraph). iv) Add a paragraph with studies showing the relationship between temperature and coral growth. v) and iv) Focus on studies from Orbicella spp. and intro to location of study as it is in Lines74 to 96.

Material and Methods

Line137 and below: More information is needed on how SST was obtained and treated. SST obtained from HadISST. I think details on whether data was downloaded as daily, monthly or annual is needed. The study specifies that annual SST was obtained, but then annual minimum and maximum SSTs were calculated. Are these annual minimum and maximum SSTs actually the minimum/maximum SST from monthly temperatures or from daily temperatures over a given year? This is not clear to me. Also the area/pixel information over which the information was obtained (i.e., resolution of data, coordinates,...). This is fundamental for reproducible science and it is missing. Make this paragraph into a new section after “Coral sampling and analysis of sclerochronological variables” along the lines of “Environmental data acquisition”.

Line 140 and throughout the text: Change annual minimal and maximal to annual minimum/maximum SST. I would recommend to thoroughly revise the grammar of the manuscript.

Lines 150 and 151: I don’t understand what the authors mean by “Only thermal anomalies and maximum SST index master were determined for their relationship with calcification rate. What is maximum SST index master? Could you explain?

Lines 154 and 155: When referring to an “index master” of sclerochronological characteristics, do the authors mean a “master chronology”? The term index master is confusing. Maybe calling it “master index” instead would make more sense, grammatically.

Line 157: When describing the master dataset being sequentially averaged 3 years, do the authors mean they applied a moving average with a 3-year window? If so, please specify to address it with the correct statistical term.

Line 158: Start a new sentence after the “averaged 3 years”. In this new sentence you describe how you calculate growth anomalies from the master dataset. This is independent from the 3-year averaging and by being in a different sentence it is clearer for the reader.

Statistical analysis and results

Not all statistical analysis explored in the results section are included in the Methods and other seems to be not well clarified. Here, I follow the structure of the results sections and detail what needs to be addressed.

Lines 166 to172: Presumably differences across time on each growth parameter were calculated with One-way ANOVA/F-test and/or Kruskal-Wallis/H-test? This is nowhere specified on the statistical analysis in methods.

Lines 174 to 183: Relationships between sclerochronological parameters are reported as R^2^ yet in methods it is described as “The relationship between the sclerochronological characteristics (skeletal density, extension rate, and calcification rate) was evaluated using simple correlations” (Lines 145-147).

Simple correlations only measure the level of association/linearity between two variables (without assigning dependency to these variables) and it is reported as “r” usually. Some examples of correlations would be (Pearson’s, Spearman’s or Kendall’s). The authors fail to describe what simple correlation method they use for these variables.

In addition, the reporting of R^2^ suggest that they might have used a simple lineal regression instead.

My preference in this case would be to use a correlation method (rather than linear modelling), because this way it is not necessary to assign what growth variable is dependent and which one is independent. For example, is linear extension dependent on skeletal density?. However, relationships between growth variables in the literature have been reported following either statistical approach, and one is no inherently wrong. But authors need to be consistent and detail what test was performed and report accordingly.

Line 185: “Index master chronological” is confusing. Maybe changing by “Master chronological index” would aid in clarity throughout the manuscript.

Lines 188: The authors describe a significant decreasing trend in density of O. faveolata. What statistical test was performed here? It is not described in the statistics method sections. A linear regression of growth variables vs. time would suffice to justify the significancy of the trends described here.

Discussion

Line 197: Add more references to this first sentence for a more compelling and strong affirmation.

Line 203: Do the authors mean here positive thermal anomalies? As these can be negative too, but the data seems to support the effect of positive maximum SST anomalies over calcification change. Please, clarify.

Line 215: Denser skeletons make colonies more susceptible to bioerosion and Line 237-238: fragile skeletons (due to higher extension and reduced density) and more susceptible to bioerosion. These two sentences contradict themselves and it is unclear to me whether denser skeletons are more prone to bioerosion or the opposite.

Line 249-250: “Under the context of climate predictions, which indicate that

ocean temperatures continue to rise and threaten corals.” This sentence feels out of context. Unclear what is the point the authors are trying to make.

Line 252: “The models presented herein indicate that most coral will suffer marked deterioration in the coming decades”. This is a strong prediction that cannot be deducted from the results of this study. This study confirms that thermal stress might has caused reduction in skeletal calcification of O. faveolate in the last two decade, but conclusion to future response of these massive species in other locations, and, much less, in “most coral reefs” cannot be drawn. Please, rephrase.

Figures and Data

Figures 3 and 4: Adding SD (or SE as error bars to these datapoints will be informative for the reader and it is important to report.

Figure 5: Equally, adding a 95% confidence ribbon to each time-series will also provide important information. For example, seems like in 1998 growth variability is wider than in other periods (from Table 1). This in itself is a very interesting observation that can provide extra information and context for the readers.

Data availability: It would be a good addition to include single core growth parameters as SOM. As it stand, only the data from the master chronologies, on a 3-year averaged mean, is available with the manuscript. Providing all raw data (growth parameters from each coral and SSTs) is essential to ensure reproducibility efforts.

Other data: Equally, it might be interesting to include in the SOM all linear regression model performed with calcification, linear extension and density with SST, maximum SST and minimum SST. This is not necessary and just an addition. As such, feel free to ignore.

**Minor changes:**

Line 27: Local anthropic stressors. Anthropogenic might be a more common adjective to use here instead (and also throughout the manuscript)

Line 37: the maximum SST has been increased is confusing as an indirect sentence and suggest it has been increased by someone/something. . Use a direct sentence for clarity: SST has increased.

Line 42: From living organisms rather than on living organisms.

Lines 42 to 45: This is one long sentence. Break into two or more sentences for clarity.

Line 45: Measurements would be more appropriate than measures. Technically both can be used as noun, but the former is more common. This is just a recommendation, feel free to ignore.

Line 64: “coral calcification and survival rates”.

Line 69: have become one most important threat to coral

Line 82: AND wave exposure.

Line 106: with an intra-annual SST, from 29.1°C during June to September and colder waters 23.5°C from January to April [35] This sentence needs to be re-structured and clearer.

Line 131: Include a sentence briefly describing the methods for density calculations used in Reference 38 (i.e., density measured over a 4x2 area every XX mm, or similar) so reader can understand this methods without having to swing between publications.
