# Supplementary material for: Sclerochronological characteristics of Orbicella faveolata in Cayo Arenas, a remote coral reef from the Gulf of Mexico
Source: PLoS One. 2023 Nov 14;18(11):e0293802. doi: 10.1371/journal.pone.0293802 (PMC10645327; doi:10.1371/journal.pone.0293802)
Supplement: Attachment — Submitted filename: Response to reviewers.docx [file pone.0293802.s004.docx]

Editor’s comments:

- Inclusion of a radiograph (s) as a figure representing your the three sclerochronological features as suggested by Reviewer #1 seems appropriate.

We added a figure (Fig. 3).

- Please review the data availability requirements of PLOS ONE. As noted by Reviewer #2, more data needs to be made available. This needs to be done in order for this paper to be published by PLOS ONE.

We added an Excel file of the full data as an appendix (Apendix 1) in the supplementary material.
